# Transcriptomic Analysis of the Rice White Tip Nematode, *Aphelenchoides besseyi* (Nematoda: Aphelenchoididae)

**DOI:** 10.1371/journal.pone.0091591

**Published:** 2014-03-17

**Authors:** Feng Wang, Danlei Li, Zhiying Wang, Airong Dong, Lihong Liu, Buyong Wang, Qiaoli Chen, Xiaohan Liu

**Affiliations:** College of Forestry, Northeast Forestry University, Harbin, Heilongjiang, China; BASF Plant Sciences – Cropdesign, Belgium

## Abstract

**Background:**

The rice white tip nematode *Aphelenchoides besseyi*, a devastating nematode whose genome has not been sequenced, is distributed widely throughout almost all the rice-growing regions of the world. The aims of the present study were to define the transcriptome of *A. besseyi* and to identify parasite-related, mortality-related or host resistance-overcoming genes in this nematode.

**Methodology and Principal Findings:**

Using Solexa/Illumina sequencing, we profiled the transcriptome of mixed-stage populations of *A. besseyi*. A total of 51,270 transcripts without gaps were produced based on high-quality clean reads. Of all the *A. besseyi* transcripts, 9,132 KEGG Orthology assignments were annotated. Carbohydrate-active enzymes of glycoside hydrolases (GHs), glycosyltransferases (GTs), carbohydrate esterases (CEs) and carbohydrate-binding modules (CBMs) were identified. The presence of the *A. besseyi* GH45 cellulase gene was verified by *in situ* hybridization. Given that 13 unique *A. besseyi* potential effector genes were identified from 41 candidate effector homologs, further studies of these homologs are merited. Finally, comparative analyses were conducted between *A. besseyi* contigs and *Caenorhabditis elegans* genes to look for orthologs of RNAi phenotypes, neuropeptides and peptidases.

**Conclusions and Significance:**

The present results provide comprehensive insight into the genetic makeup of *A. besseyi*. Many of this species' genes are parasite related, nematode mortality-related or necessary to overcome host resistance. The generated transcriptome dataset of *A. besseyi* reported here lays the foundation for further studies of the molecular mechanisms related to parasitism and facilitates the development of new control strategies for this species.

## Introduction

Parasitic nematodes that prey on plants cause devastating losses in agricultural production and contribute to a significant reduction in yield. It has been estimated that plant parasitic nematodes cause annual losses in crop production valued at US$125 billion [1]. The rice white tip nematode *Aphelenchoides besseyi* Christie, 1942, which is widely distributed throughout almost all the rice growing regions of the world, is considered to be a major contributor to the seed-borne pathogens of rice [2]–[4]. *A. besseyi* is a global burden, causing losses of up to 50% in upland rice in Brazil [5] and 10–50% in flooded rice in China [6].

As a seed-borne nematode, *A. besseyi* can survive on stored grain in anhydrobiosis for several years [7]. This species is a facultative migratory ecto- and endoparasite of the leaf or young tissue of rice, causing whitening of the top of the leaf, which later becomes necrotic, and a crinkling and distortion of the flag leaf enclosing the panicle, which then dies off and disintegrates [4], [8]. Moreover, *A. besseyi* is polyphagous and damages a wide range of host plants, including many important crop species, such as strawberry (*Fragaria grandiflora*), sweet corn (*Zea mays*), sweet potato (*Dioscorea esculenta*), bird's nest fern (*Asplenium nidus*) and many vegetables and grasses [9]–[11].

Based on its phylogenetic tree [12], *A. besseyi* belongs to clade 10B, whereas most other major plant-parasitic nematodes belong to clade 12. Only a high-quality draft genome sequence from *Bursaphelenchus xylophilus* is available for the investigation of the biological basis of clade 10 nematodes [13]. The investigation of *B. xylophilus* genes linked to key biological processes and parasitism will provide guidance for further studies of *A. besseyi* and the other clade 10 nematodes. Although the model free-living nematodes *Caenorhabditis* spp. (clade 9A), the human parasite *Brugia malayi* (clade 8B) and several plant-parasitic nematodes (clade 12) have been subjected to molecular analysis [14], very little is known about the genomic background of *A. besseyi*. However, recently, a fatty acid and retinoid-binding protein gene (*Ab-far-1*) was cloned from *A. besseyi* based on EST sequencing [15]. In addition, an exciting small-scale transcriptome project for *A. besseyi* was recently published [16], [17].

Next-generation sequencing technology, such as the Solexa/Illumina, 454 (Roche) and ABI-SOLiD platforms, has dramatically improved the efficiency of gene discovery [18]. Using next-generation sequencing technology, several nematode transcriptomes have been sequenced, including *Trichuris suis*, *Necator americanus*, *B. malayi*, *Trichostrongylus colubriformis*, *Pratylenchus coffeae* and *P. thornei* [14], [19]–[24]. Whereas genomic analyses of *B. xylophilus* reveal the genetic potential of clade 10 nematodes, transcriptomic studies of nematodes will enable the regulatory networks controlling the expression of parasitism-related genes in space and at different stages to be deciphered.

In the present study, Solexa/Illumina sequencing technology was used to explore and functionally annotate the transcriptome of a mixed-stage population of *A. besseyi*. The results provide insights into the genetic background of *A. besseyi*. The transcriptomic data presented here serve as a valuable resource that will help deepen our understanding of the parasitism of this nematode. Understanding the basic biology of plant-parasitic nematodes, especially the parasitic mechanisms at the molecular level, is essential for the development of effective control strategies.

## Materials and Methods

### Nematode Material

The nematode *A. besseyi* (NCBI BioSample accession No. SAMN02420038), derived from a single female isolated from an infected rice grain in eastern China, was maintained by serial subculture on a *Botrytis cinerea* mat grown on sterilized barley [25] and kept in the dark at 25°C. Nematodes freshly extracted from sterilized barley were used for RNA extraction. Eggs, juvenile stages (J2–J4) and adults were mixed together at a ratio of 1∶2∶3.

### RNA Extraction, cDNA Library Construction and Illumina Sequencing

Total RNA was extracted from mixed-stage nematodes using TRIzol regent (Invitrogen, Carlsbad, CA, USA) [26] and then cleaned using an RNeasy Minikit column (Qiagen, Valencia, CA, USA) according to the manufacturers' instructions. The extracted RNA was assessed for quality and quantified using a BioPhotometer D30 (Eppendorf, Hamburg, Germany).

Two micrograms of total RNA was used to extract polyadenylated RNA and construct strand-specific RNAseq libraries according to the Illumina protocol (directional mRNASeq sample preparation part #15018460 Rev. A, October 2010; Illumina, San Diego, CA, USA) (detailed in Text S1 in File S1). The library quality was validated with a Bioanalyzer 2100 (Agilent, CA, USA). Each sample was sequenced for 80 cycles on one lane of the Illumina GAIIX platform (Illumina, San Diego, CA, USA) with a 2×100 bp module by LC Sciences (Houston, TX, USA). The dataset generated by deep-sequencing platforms was deposited in the NIH short-read archive (accession No. SRR1040470).

### Data Filtering and *De Novo* Assembly

Low-quality reads with ambiguous sequences “N” were removed according to a sliding window method using a threshold of 35 bp. To examine the coverage of the sequences, 500,000 randomly selected reads were compared with the NT (non-redundant nucleotide sequences in the NCBI) database using BLAST, and a typical cutoff expectation value (E-value) was less than 1e-10. The trinity (trinityrnaseq_r2013-02-25) [27] and paired-end methods were used to generate non-redundant unigenes via the *de novo* assembly of the clean reads. The quality of the assembly was critically assessed by LC Sciences (Houston, TX, USA) before subsequent analysis.

The expression level of each transcript was measured as the number of clean reads mapped to its sequence. The mapped clean read number was normalized to RPKM (reads per kilobase of exon model per million mapped reads) with Bowtie 0.12.8 (single-end mapping) and adjusted with a normalized factor [28].

To determine how many unigenes in total were available in our transcriptome and another recently published *A. besseyi* transcriptome [16], we compared the unigenes with the 5,804 MIRA assembly sequences downloaded from nematode.net [29] using BLASTN with an E-value cutoff of 1e-6.

### Annotation and KEGG Pathway Determination

Unigene sequence directions were determined by searches against the NR (non-redundant protein sequences in the NCBI), Swiss-Prot, TrEMBL, Cdd, Pfam and KOG databases (E-value<1e-5).

KEGG mapping was used to determine the metabolic pathways of metabolism, genetic information processing, environmental information processing, cellular processes, organismal systems and *etc.* [30]. Sequences were submitted to the KEGG Automatic Annotation Server (KAAS) [31] to enrich the pathway annotation.

### Annotation of Carbohydrate-Active Enzymes

The CAZymes Analysis Toolkit [32] was used to detect *A. besseyi* carbohydrate-active enzymes (CAZymes) according to a previous report [13]. Expansin-like proteins, pectinases and arabinanases were detected with a BLAST search using core modules of known protein sequences as queries. BLASTP was used to predict protein functions by searching against known protein modules and catalytic sites downloaded from NCBI's Conserved Domain Database service. Multiple sequence alignments were performed using Clustal W, and phylogenetic trees were constructed based on those sequences using the MEGA 5.05 program.

### Effector Candidate Identification

Known putative effectors and selected annotated secreted protein sequences from different plant-parasitic nematodes (*Aphelenchus avenae*, *B. mucronatus*, *B. xylophilus*, *Ditylenchus africanus*, *Globodera mexicana*, *G. pallida*, *G. rostochiensis*, *G. tabacum*, *Heterodera glycines*, *H. schachtii*, *M. arenaria*, *M. chitwoodi*, *M. hapla*, *M. incognita*, *M. javanica*, *P. coffeae*, *P. penetrans*, *Rotylenchulus reniformis*, *Radopholus similis* and *Xiphinema index*) [33] were downloaded from GenBank. These sequences were searched against the *A. besseyi* contigs with TBLASTN to identify potential homologs [34]. An E-value cutoff of 1e-5 was used to identify significant matches. SignalP 4.1 [35] and TMHMM 2.0 [36] were used to identify secreted proteins as previously described [13].

### RNAi Phenotype Matches to *C. elegans* Genes

Seventy-five *C. elegans in vitro* RNA interference (RNAi)-related genes were identified and retrieved from WormBase (version WS236). The *A. besseyi* contigs were compared with *C. elegans* genes to identify orthologs of RNAi phenotypes [37].

### Neuropeptides and Peptidase Detection

One hundred thirteen *C. elegans* neuropeptide genes were identified and retrieved from WormBase (version WS236). The *A. besseyi* contigs were compared with *C. elegans* genes to identify orthologs with high identity to neuropeptides by BLAST.

The MEROPS database 9.8 was used to identify putative *A. besseyi* peptidases [38]. The candidates were derived and examined for similarity (E-value<1e-10) to MEROPS proteins.

### *In Situ* Hybridization of GH45 Cellulases

*In situ* hybridization was performed on *A. besseyi* essentially as described by De Boer *et al.* [39]. A digoxigenin-labeled *A. besseyi* GH45 cellulase DNA probe was synthesized using the cDNA sequence following the general protocol of the Roche DIG DNA Labeling Kit (Roche, Mannheim, Germany, cat. no. 11175033910). The specificity of the probe was verified by Southern blotting. The hybridization signals were detected by incubation with the DIG High Prime DNA Labeling and Detection Starter Kit I (Roche, Mannheim, Germany, cat. no. 11745832910) and photographed under a Bx51 microscope (Olympus, Tokyo, Japan).

## Results

### Illumina Sequencing and *De Novo* Assembly

A total of 46,826,350 raw reads were obtained by Solexa (Illumina GAIIX) RNA paired-end sequencing, and 36,905,372 high-quality clean reads were obtained after a strict filtering process. The average length of the clean reads was 89.89 bp. The ratio of clean reads was 78.81%. No RNA contamination from *B. cinerea* or other organisms was found based on 500,000 randomly selected reads from the NT database. Using the clean reads, Trinity produced 51,270 transcripts with length >200 bp. The average length and N50 of these transcripts were 1,241 bp and 1,857 bp, respectively. A total of 35,180 unigenes with an average length of 1,050 bp and N50 of 1,626 bp was generated after further clustering and assembly. Of these unigenes, 21,882 unigenes (62.2%) were >500 bp and 13,259 unigenes (37.7%) were >1,000 bp (Figure S1 in File S1). The frequency distribution of the GC content is shown in Figure S2 in File S1.

Recently, another *A. besseyi* transcriptome including 5,804 MIRA assembly sequences was published [16]. Compared with this transcriptome (detailed in the supplementary dataset in File S2), 77.8% (27,380 out of 35,180) of the unigenes in our transcriptome were unique. Only 2.2% (125 out of 5,804) of the MIRA assembly-yielded sequences were not found in our dataset (detailed in Text S2 in File S1). Because the E-value cutoff of 1e-6 was used, 7,800 (22.2% of the total 35,180) of the unigenes in our transcriptome matched 5,679 (97.8% of the total 5,804) MIRA assembly sequences. Therefore, at least 33,184 unigenes are now available in the two datasets combined.

The distribution of transcript values indicated that all transcripts were expressed, and 95.69% of the reads were mapped (Table S1 in File S1). Of the 51,270 transcripts, the RPKM values of the majority of the transcripts varied from 1 to 12 (Table S2 and Figure S3 in File S1). The distribution of RPKM values indicated that most genes were expressed at low levels.

### Annotation

**Figure 1:**
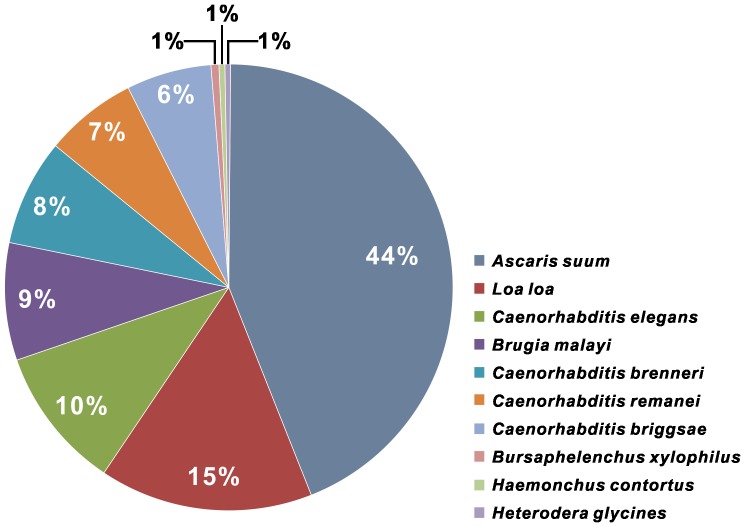
The ten most common NR hierarchies identified in the *A. besseyi* sequences.

**Figure 2:**
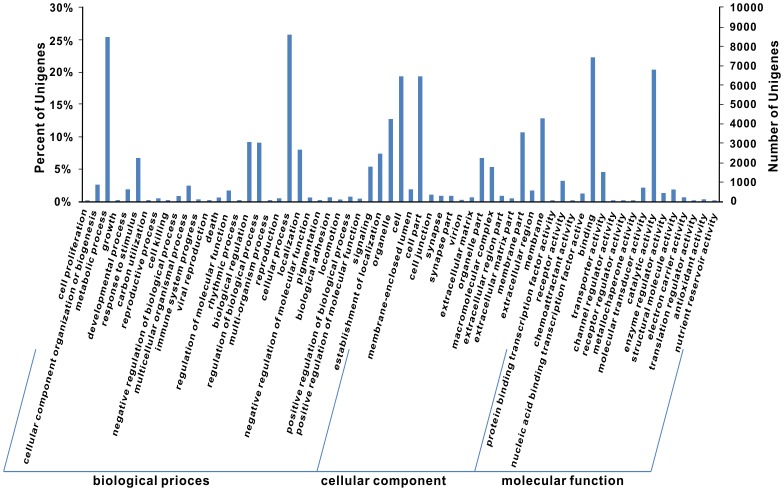
The most abundant Gene Ontology terms present in the dataset for the cellular component, molecular function and biological process categories.

Unigenes were determined by performing BLASTX searches against the NR protein database. A total of 17,338 orthologs were identified. Of the 35,180 unigenes, 88.03% were annotated as the most common ten organisms (Figure 1). The majority of the annotated *A. besseyi* (clade 10B) sequences corresponded to known nucleotide sequences of nematodes belonging to clades 2A (*Trichinella spiralis*), 8B (*Ascaris suum*, *Loa loa* and *B. malayi*), 9A (*Caenorhabditis* spp.) and 10B (*B. xylophilus*). Other protein databases, such as Swiss-Prot, TrEMBL, Cdd, Pfam and KOG, were used to annotate these unigenes (detailed in Table S3 in File S1). A search for Gene Ontology (GO) identifiers for these unique sequences was performed, and 14,064 GO terms were retrieved. The most abundant GO terms in the dataset are shown in Figure 2.

### Pathway Determination

**Figure 3:**
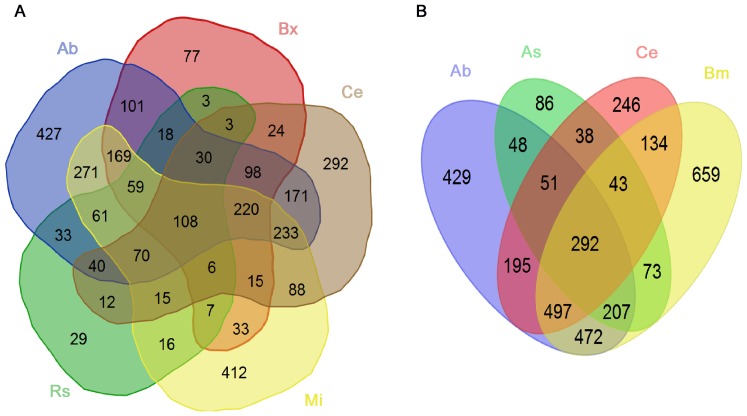
The intersections of lists of nematode KEGG Orthology (KO) assignments. A: Assignment of *C. elegans*, *A. besseyi* and other plant-parasitic nematodes. B: Assignment of *C. elegans*, *A. besseyi* and the animal-parasitic nematode *A. suum*. Ab: *A. besseyi*, Bx: *B. xylophilus*, Mi: *M. incognita*, Ce: *C. elegans*, Bm: *B. malayi*, As: *A. suum*.

KAAS was used to annotate the EST sequences of the animal nematodes (*B. malayi* and *A. suum*), the free-living nematode (*C. elegans*), *A. besseyi* and other plant-parasitic nematode (*B. xylophilus* and *M. incognita*) transcripts. Of all the *A. besseyi* transcripts, 9,132 KEGG Orthology (KO) assignments were annotated. Two Venn diagrams were drawn to show the intersections of the nematode KO assignments (Figure 3). The diagram of *C. elegans*, *A. besseyi* and the other plant parasitic nematodes indicated 8,705 *A. besseyi* KO assignment hits in one or more of the nematodes, of which 427 KO assignments were unique (Figure 3A). Another diagram of *C. elegans*, *A. besseyi* and the animal-parasitic nematode *A. suum* also illustrates 429 unique *A. besseyi* KO assignments (Figure 3B).

To identify the pathways in which the unigenes participated, we mapped the *A. besseyi* unigenes to the canonical reference pathways in the KEGG database. A total of 9,133 unigenes were annotated based on a BLASTX search of the KEGG database; 753 enzymes and 306 KEGG pathways were predicted (Table 1, Tables S4–S8 in File S1). Of these KEGG pathways, 121 pathways were consistent with the corresponding pathways in *C. elegans*. In addition to the most common 5 pathways (Table 2), other important pathways, including ubiquitin-mediated proteolysis (72 members), glutathione metabolism (39 members), glycolysis/gluconeogenesis (38 members), glycerophospholipid metabolism (38 members), drug metabolism (35 members) and metabolism of xenobiotics by cytochrome (32 members), were found.

**Table 1:** The classification of *A. besseyi* pathways.

	Pathways	Members
**Metabolism**	121	2077
**Genetic Information Processing**	22	868
**Environmental Information Processing**	25	478
**Cellular Processes**	17	583
**Organismal Systems**	60	957
**Human Diseases**	61	1396
**Total**	306	4489

**Table 2:** The most common 5 *A. besseyi* pathways consistent with *C. elegans*.

Pathway	Pathway type	Unigene num
**cel01100**	Metabolic pathways	602
**cel03010**	Ribosome	119
**cel04141**	Protein processing in endoplasmic reticulum	118
**cel00190**	Oxidative phosphorylation	106
**cel03013**	RNA transport	99

### CAZymes and Cell Wall Degradation

Some CAZymes are known to be secreted by sedentary as well as migratory plant parasitic nematodes and have been predicted to be involved in nematode migration in the plant and feeding from host cells [40]. According to the Carbohydrate-Active enZYmes Database, CAZymes are grouped into the following classes: glycoside hydrolases (GHs), glycosyltransferases (GTs), polysaccharide lyases (PLs), carbohydrate esterases (CEs) and carbohydrate-binding modules (CBMs) [24]. A summary of the CAZymes detected in *A. besseyi* is provided in Table 3. In all, 524 contigs were assigned to the following four classes of CAZymes: 136 contigs with GH domains to 10 families of GHs (Table S9 in File S1), 328 contigs to 28 families of GTs (Table S10 in File S1), 42 contigs to 2 families of CEs (Table S11 in File S1) and 18 contigs to 3 families of CBMs (Table S12 in File S1). Apparently, PLs are not present in any of the contigs in this transcriptome.

**Table 3:** Carbohydrate-active enzymes identified in *A. besseyi*.

CAZy enzyme classes	CAZy family	*A. besseyi* contigs
**Glycoside hydrolases**	10	136
**Glycosyl transferases**	28	328
**Carbohydrate esterases**	2	42
**Polysaccharide lyases**	0	0
**Carbohydrate-binding modules**	3	18
**Total**	43	524

**Figure 4:**
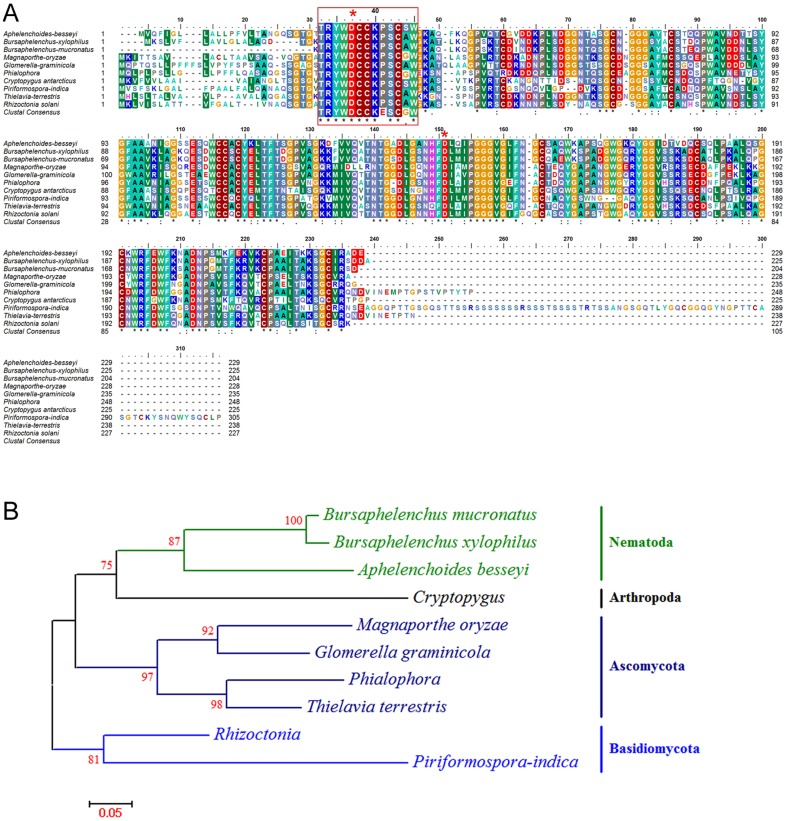
Alignment and polygenetic tree of the *A. besseyi* GH45 cellulase amino acid sequence with its homologs identified from the NCBI database. A: The alignment shows the comparison between *A. besseyi* GH45 cellulase and its homologs. Active site residues are marked with red asterisks. The GH45 cellulase consensus sequences are boxed with a red line and highlighted with a magnifying lens. Asterisks, double dots and single dots denote fully conserved, strongly conserved and weakly conserved amino acid residues, respectively. B: The maximum likelihood tree shows the relationship between *A. besseyi* GH45 cellulase and homologs. One thousand bootstrap replicates were performed, and the node labels represent the percentage of bootstrap support.

With regard to plant cell wall degradation-related CAZymes, we first noted that 4 contigs were identified as homologs of GH45 cellulases previously identified in *B. xylophilus* and another *A. besseyi* transcriptome [41]. Multiple sequence alignments of *A. besseyi* GH45 cellulase with *B. xylophilus* homologs and those of Ascomycota and Basidiomycota fungi revealed that the motifs were almost identical in all proteins (Figure 4A). The GH45 cellulase maximum likelihood tree (Figure 4B) showed four major clusters. Because no other nematode GH45 cellulases could be found, *B. xylophilus* and *B. mucronatus* were the only two plant parasitic nematodes used in the phylogenetic tree study in addition to *A. besseyi*.

**Figure 5:**
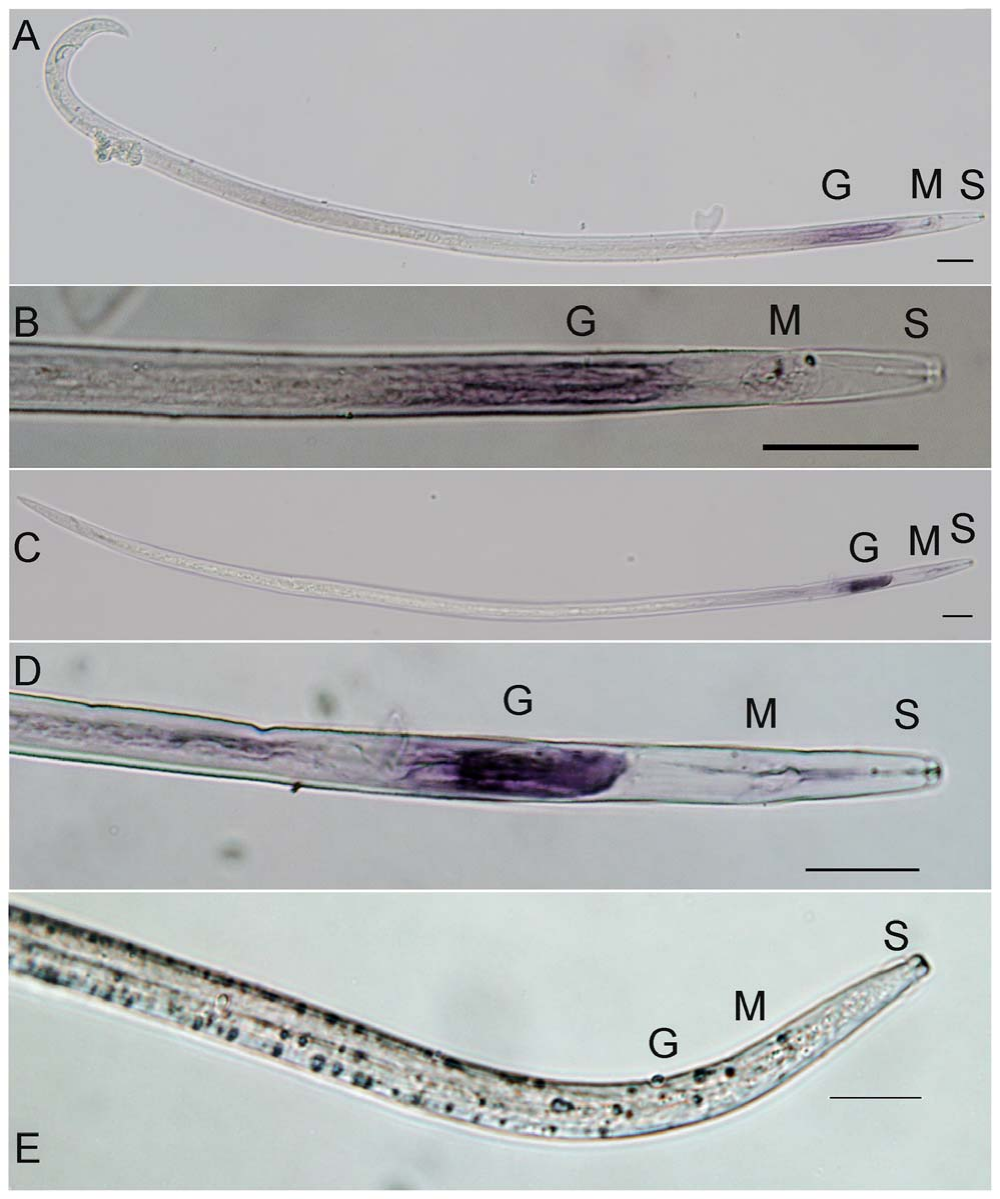
Localization of *A. besseyi* GH45 cellulase mRNA by *in situ* hybridization using digoxigenin-labeled antisense or sense probes. A and B: Antisense probe in a male nematode. C and D: Antisense probe in a female nematode. E: Control sense probe in a female nematode. The scale bars represent 20 µm. G: esophageal glands; S: stylet; M: metacarpus.

*In situ* hybridization was used to determine the tissue specificity of *A. besseyi* GH45 cellulase gene transcription. The anti-sense probe stained the esophageal gland cell area (Figure 5). The gland region was strongly stained by probes against the *A. besseyi* GH45 cellulase gene, whereas the sense probes gave no signal (Figure 5E). The *A. besseyi* GH45 cellulase gene was expressed at a lower level in males (Figure 5A and B) than in females (Figure 5C and D).

Another plant cell wall degradation-related CAZyme family secreted by plant parasitic nematodes is GH5 cellulase, which has been well studied in the plant-parasitic nematodes of the order Tylenchida (clade 12, including most of the major plant-sedentary endoparasitic nematodes such as *Meloidogyne* spp., *Heterodera* spp. and *Globodera* spp. and the migratory parasitic species *P. thornei* and *P. coffeae*) [14], [24]. However, GH5 cellulases were apparently absent from the *A. besseyi* transcriptome. Furthermore, GH28 polygalacturonase, GH30 xylanase, GH43 arabinase, GH53 arabinogalactan galactosidase, PL3 pectate lyase and expansin-like protein were absent.

In addition to plant cell wall degradation-related CAZymes, we also noted that *A. besseyi* possessed GH16 and GH20 CAZymes, which may be involved in fungal cell wall degradation (Table S9 in File S1). GH16, which degrades beta-1,3-glucan, a core component of the fungal cell wall, was present in this *A. besseyi* transcriptome in remarkable abundance (a total of 90 contigs). In contrast, GH20, which is involved in chitin degradation, was detected in low abundance (2 contigs).

### Candidate Effector Identification

Numerous effectors with various functions in the plant cell are secreted into the plant by nematodes [33]. Unlike sedentary or migratory endoparasites, *A. besseyi* is a migratory ecto- and endoparasite nematode that does not rely on biotrophy. Candidate *A. besseyi* parasitism genes and effector homologs were identified to establish the relationships between the parasite strategy and parasitism genes. All known and annotated putatively secreted proteins from plant-parasitic nematodes were compared with the *A. besseyi* contigs to identify possible homologs.

In addition to the CAZymes described above, 41 candidate effector homologs were identified (Table S13 in File S1). Thirteen unique *A. besseyi* potential effector genes were identified (Table 4) from the 41 candidate effector homologs based on the defining characteristic that effector genes that encode proteins have a putative signal peptide but lack a transmembrane helix and have an open reading frame of at least thirty amino acids after the predicted signal peptide cleavage site [42]–[44]. In contrast to sedentary endoparasites, no plant-hormone mimics or nodulation-related effector homologs were found in the *A. besseyi* transcriptome.

**Table 4:** Potential effectors identified in *A. besseyi*.

Effector	Function	Contigs with hit	Bit score	Best E-value
**4D01**	unknown	21	135	2e-32
**5G05**	unknown	6	175	5e-44
**6F06**	unknown	56	66	5e-11
**Acid phosphatase**	digestion	30	324	1e-88
**Calreticulin**	calcium signaling	24	617	e-177
**Cathepsin**	protein degradation	2	449	e -126
**Chitinase**	egg hatching	7	125	9e-29
**C-type lectin**	unknown	3	48	4e-05
**FAR**	binding of host fatty acids; reducing defense response	12	177	4e-45
**Glutathione peroxidase**	detoxification of ROS	10	360	e-100
**PDI**	protein folding	4	706	0
**Putative esophageal gland cell secretory protein 3**	unknown	10	135	2e-32
**SXP-RAL2**	unknown	2	105	1e-23

Because *A. besseyi* does not establish a long-term feeding relationship with the living cells of their hosts, it was necessary to confirm the 13 potential effectors by functional classification. Similar to *B. xylophilus* [13], the majority (7 out of the 13) of *A. besseyi* candidate effectors were also present in other migratory plant nematodes, *C. elegans* and even animal-parasitic nematodes. Cathepsin-like proteinases were also found in another migratory nematode, *R. similis* [45]. Chitinase, an egg-hatching-related protein, was found in *Caenorhabditis* spp. and *L. loa*. Glutathione peroxidase was found in *Caenorhabditis* spp., *A. suum* and *Haemonchus contortus*. FAR, which was also found in an *A. besseyi* EST project, was found in *Caenorhabditis* spp. and *H. contortus*. PDI was found in *Caenorhabditis* spp., *B. malayi* and *L. loa*. The other 6 effector homologs had unknown functions. Given that all the identified genes are previously assumed to be involved in the process of syncytia or giant cell formation, these findings suggest that the roles of these genes may be different in *A. besseyi*.

### The Presence of Genes Involved in the RNAi Pathway

RNAi is an effective method for gene function identification and will facilitate the identification of potential targets for developing potent antinematodal drugs against *A. besseyi*. RNAi has been successfully applied in both sedentary and migratory plant-parasitic nematodes, including more than twenty plant-parasitic nematodes [46]–[50]. Recently, RNAi was successfully applied to *A. besseyi* [15].

A comparative analysis was conducted with the *A. besseyi* contigs and *C. elegans* genes to look for RNAi phenotype orthologs. The 2,899 *A. besseyi* contigs homologous to *C. elegans* matched 57 RNAi pathway effectors. The RNAi-related proteins were separated into the following five core functional groups: small RNA biosynthesis, dsRNA uptake and spreading, argonautes (AGOs) and RNA-induced silencing complex (RISC), RNAi inhibitors and nuclear effectors (Table 5 and Tables S14–S18 in File S1). Apparently, *A. besseyi* encodes more predicted RNAi pathway effectors than *B. xylophilus* [13] and *M. incognita* [36].

**Table 5:** Presence of genes involved in the RNAi pathway.

	Number of RNAi effector proteins				*A. besseyi* contigs*
	*A. besseyi*	*C. elegans*	*B. xylophilus*	*M. incognita*	
	clade 10B	clade 9A	clade 10B	clade 12B	
**Small RNA biosynthesis**	7	9	8	7	112
**dsRNA uptake and spreading**	6	11	6	4	84
**AGOs and RISC**	27	30	12	9	2212
**RNAi inhibitors**	5	10	2	2	73
**Nuclear effectors**	12	15	9	6	418
**Total**	57	75	37	28	2899

*(bitscore>40 and E-value<e-4).

### Neuropeptide Detection

Neuropeptides play important roles in the modulation of synaptic activity and the development of the nervous system and may also function as primary neurotransmitters [24], [51]. According to WormBook, 113 *C. elegans* neuropeptide genes, which encode over 250 distinct neuropeptides, have been identified [52].

With the sequencing of the *A. besseyi* transcriptome, 49 neuropeptide genes were identified (Table 6). Of these, 8 genes encode insulin-like peptides (Table S19 in File S1), 17 genes encode FMRFamide-related peptides (Table S20 in File S1) and 24 genes encode non-insulin or non-FLP peptides (Table S21 in File S1).

**Table 6:** Neuropeptide genes in *A. besseyi*.

Neuropeptide	Genes	Contigs with hit	E-value level
**Insulin-like peptides**	8	25	0.003-8e-015
**FMRFamide-related peptides**	17	54	5e-005-6e-028
**Non-insulin, non-FLP peptides**	24	288	3e-005-2e-032
**Total**	49	367	

### Peptidase Detection

The MEROPS database classifies peptidases into seven catalytic types: aspartic, glutamic, cysteine, isoleucine, metallo, serine and threonine peptidases. In our analysis, 73 peptidase genes (Table 7 and Table S22 in File S1) were identified in *A. besseyi*. Proteins belonging to the C01A (41 contigs), I02 (89 contigs), M12A (43 contigs) and S09X (110 contigs) families were particularly expanded in *A. besseyi*. Peptidase families involved in extracellular digestion and lysosomal activities were also particularly expanded.

**Table 7:** Summary of peptidases in *A. besseyi*.

Catalytic types	Family number	Best E value
**Aspartic**	2	1.50E-106
**Cysteine**	19	1.60E-108
**Isoleucine**	6	1.10E-55
**Metallo**	26	1.50E-148
**Serine**	16	9.60E-100
**Threonine**	3	5.50E-202
**Unassigned**	1	2.10E-46
**Total**	73	

## Discussion

This study provides the first detailed description of the transcriptome of *A. besseyi*, a rice-parasitic nematode without a sequenced genome. We detected and annotated the repertoire of candidate CAZymes in the *A. besseyi* transcriptome. Our dataset extends the knowledge of the arsenal of enzymes in *A. besseyi*. Cellulases are classified into 14 glycoside hydrolase families—GH5, 6, 7, 8, 9, 10, 12, 26, 44, 45, 48, 51, 61 and 74 [53] —most of which are typically produced by plant pathogens and plant parasites [54]. GH45 cellulases are present in *Bursaphelenchus* species and *A. besseyi* [13], [16]. The discovery of these genes led to the hypothesis that they were acquired from fungi via horizontal gene transfer (HGT) [40].

Although a GH5 cellulase was found in *A. fragariae* [55], no transcripts similar to GH5 cellulases were present in the *A. besseyi* transcriptome. To confirm this result, a TBLASTN search of *A. fragariae* GH5 cellulase protein sequences (NCBI accession Nos. AFD33557, AFD33558 and AFI63769) against the *A. besseyi* contigs was performed, but no hits were found with an E-value cutoff of 1e-4. It is worth noting that a small-scale transcriptome project for *A. besseyi* has been published [16]. GH45 cellulase was found in this transcriptome. However, no transcripts similar to GH5 cellulases were identified in the small-scale transcriptome. These results are consistent with our study.

In our work, a remarkable abundance of GH16 (endo-1, 3-beta-glucanase), which is thought to be important for the degradation of fungal cell walls, was detected. This enzyme has been identified in various fungivorous and plant-parasitic nematodes, first in *Bursaphelenchus* species [56] and more recently in *A. avenae* [57]. Because it is a facultative fungal feeder, it makes sense that *A. besseyi* harbors considerable GH16. This result supports the possibility that these fungi-consuming nematodes acquired endo-1,3-β-glucanase genes from bacteria to obtain their fungal feeding ability and subsequently acquired cellulase genes from fungi, which permitted them to parasitize plants [56], [58].

Of particular interest was the detection of candidate effector homologs such as cytokinins, ubiquitin extension protein, calreticulin, 10A06 and NULG1a [59] (Table S13 in File S1), although these genes were thought to be involved in the process of either syncytia or giant cell formation [47], [60]–[63]. Because *A. besseyi* does not generate specific feeding cells, the roles of these genes may indicate a different function. In addition, the effector identification of the small-scale transcriptome project for *A. besseyi* indicated that almost all (12 out of 13) of the candidate sequences had matches in *Caenorhabditis elegans* and in other nematodes [16]. These results suggested that whether the identified *A. besseyi* homologs really play the same roles as the effectors of root knot and cyst nematodes in parasitism is an open question. Obviously, further studies of these homologs are merited.

The present results provide insights into the genetic background of *A. besseyi*. These insights will be useful in future investigations of nematode parasitism the development of techniques for nematode control and plant protection.

## Supporting Information

File S1 **Supporting Figures, Tables, and Text.** Figure S1, Length distribution of All-Unigene. Figure S2, GC content frequencies distribution. Figure S3, RPKM distribution of transcript. Table S1, Expressed transcripts. Table S2, RPKM of the *A. besseyi* transcripts. Table S3, The annotation of *A. besseyi* unigenes. Table S4, Metabolism related pathways. Table S5, Genetic information processing related pathways. Table S6, Environmental information processing related pathways. Table S7, Cellular processes related pathways. Table S8, Organismal systems related pathways. Table S9, Glycoside hydrolases identified in *A. besseyi*. Table S10, Glycosyl transferases identified in *A. besseyi*. Table S11, Carbohydrate esterases identified in *A. besseyi*. Table S12, Carbohydrate binding modules identified in *A. besseyi*. Table S13, Candidate effectors or potential parasitism genes identified in *A. besseyi*. Table S14, Small RNA biosynthesis proteins. Table S15, dsRNA uptake and spreading effectors. Table S16, Argonautes and RNA-induced silencing complex components. Table S17, RNAi inhibitors. Table S18, Nuclear effectors. Table S19, Neuropeptide genes encoding insulin-like peptides. Table S20, Neuropeptide genes encoding FMRFamide-related peptides. Table S21, Neuropeptide genes encoding non-insulin, non-FLP peptides. Table S22, Peptidases identified in *A. besseyi*. Text S1, Detailed methods of RNAseq libraries construction. Text S2, MIRA assembly yielded sequences were not found in our dataset.
(DOC)

Files S2 **Supplementary Dataset.** The comparison of unigenes with another *A. besseyi* transcriptome.
(XLSX)
