# Supplementary material for: Transcriptomic Analysis of the Rice White Tip Nematode, Aphelenchoides besseyi (Nematoda: Aphelenchoididae)
Source: PLoS One. 2014 Mar 17;9(3):e91591. doi: 10.1371/journal.pone.0091591 (PMC3956754; doi:10.1371/journal.pone.0091591)
Supplement: File S1 — Supporting Figures, Tables, and Text. Figure S1, Length distribution of All-Unigene. Figure S2, GC content frequencies distribution. Figure S3, RPKM distribution of transcript. Table S1, Expressed transcripts. Table S2, RPKM of the A. besseyi transcripts. Table S3, The annotation of A. besseyi unigenes. Table S4, Metabolism related pathways. Table S5, Genetic information processing related pathways. Table S6, Environmental information processing related pathways. Table S7, Cellular processes related pathways. Table S8, Organismal systems related pathways. Table S9, Glycoside hydrolases identified in A. besseyi. Table S10, Glycosyl transferases identified in A. besseyi. Table S11, Carbohydrate esterases identified in A. besseyi. Table S12, Carbohydrate binding modules identified in A. besseyi. Table S13, Candidate effectors or potential parasitism genes identified in A. besseyi. Table S14, Small RNA biosynthesis proteins. Table S15, dsRNA uptake and spreading effectors. Table S16, Argonautes and RNA-induced silencing complex components. Table S17, RNAi inhibitors. Table S18, Nuclear effectors. Table S19, Neuropeptide genes encoding insulin-like peptides. Table S20, Neuropeptide genes encoding FMRFamide-related peptides. Table S21, Neuropeptide genes encoding non-insulin, non-FLP peptides. Table S22, Peptidases identified in A. besseyi. Text S1, Detailed methods of RNAseq libraries construction. Text S2, MIRA assembly yielded sequences were not found in our dataset. (DOC) [file pone.0091591.s001.doc]

# File S1


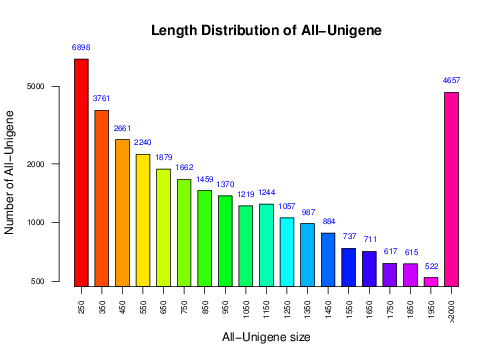


**Figure S1 Length distribution of All-Unigene**


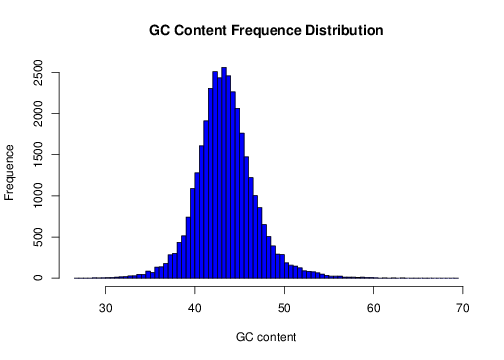


**Figure S2 GC content frequencies distribution**


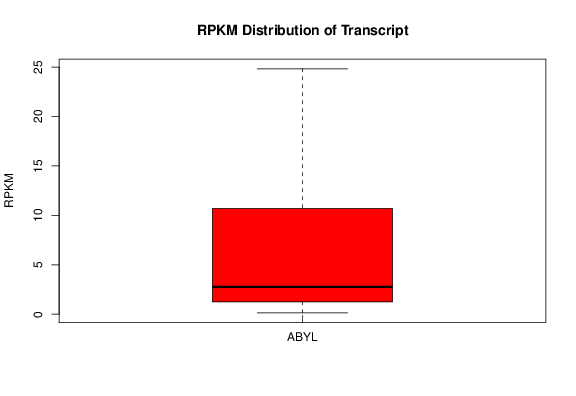


**Figure S3 RPKM distribution of transcript**

**Table S1 Expressed transcripts**

| **Valid data** | **Map data** | **Data%** | **All transcript** | **Exp transcript** | **Transcript%** |
| --- | --- | --- | --- | --- | --- |
| **36,905,372** | 35,316,337 | 95.69% | 51,270 | 51,270 | 100.00% |

**Table S2 RPKM of the *A. besseyi* transcripts**

| **Exp transcript** | **Min.** | **1^st^ Qu.** | **Median** | **Mean** | **3^rd^ Qu.** | **Max.** | **Sd.** | **Sum.** |
| --- | --- | --- | --- | --- | --- | --- | --- | --- |
| **51,270** | 0.14 | 1.25 | 2.76 | 16.51 | 10.68 | 7,165.54 | 88.44 | 846287.94 |

**Table S3 The annotation of *A. besseyi*** unigenes

| **Unigene num** | **NR** | **SWISS-PROT** | **TREMBL** | **CDD** | **PFAM** | **KOG** |
| --- | --- | --- | --- | --- | --- | --- |
| **35180** | 17338 | 10997 | 17727 | 12312 | 17157 | 9306 |
|  | 49.28% | 31.26% | 50.39% | 35.00% | 48.77% | 26.45% |

**Table S4** Metabolism related pathways

|  | **Pathways** | **Members** |
| --- | --- | --- |
| **Global map** | 3 | 792 |
| **Carbohydrate metabolism** | 15 | 229 |
| **Energy metabolism** | 8 | 173 |
| **Lipid metabolism** | 16 | 143 |
| **Nucleotide metabolism** | 2 | 134 |
| **Aminoacid metabolism** | 13 | 196 |
| **Metabolism of other amino acids** | 7 | 51 |
| **Glycan biosynthesis and metabolism** | 13 | 122 |
| **Metabolism of cofactors and vitamins** | 12 | 74 |
| **Metabolism of terpenoids and polyketides** | 8 | 26 |
| **Biosynthesis of other secondary metabolites** | 11 | 26 |
| **Xenobiotics biodegradation and metabolism** | 11 | 65 |
| **Reaction module maps** | 2 | 46 |
| **Chemical structure transformation maps** | 0 | 0 |
| **Total** | 121 | 2077 |

**Table S5** Genetic information processing related pathways

|  | **Pathways** | **Members** |
| --- | --- | --- |
| **Transcription** | 3 | 151 |
| **Translation** | 5 | 333 |
| **Folding, sorting and degradation** | 7 | 269 |
| **Replication and repair** | 7 | 115 |
| **Total** | 22 | 868 |

**Table S6 Environmental information processing related pathways**

|  | **Pathways** | **Members** |
| --- | --- | --- |
| **Membrane transport** | 2 | 12 |
| **Signal transduction** | 19 | 427 |
| **Signaling molecules and interaction** | 4 | 39 |

**Table S7** Cellular processes related pathways

|  | **Pathways** | **Members** |
| --- | --- | --- |
| **Transport and catabolism** | 5 | 202 |
| **Cell motility** | 1 | 46 |
| **Cell growth and death** | 7 | 204 |
| **Cell communication** | 4 | 131 |

**Table S8** Organismal systems related pathways

|  | **Pathways** | **Members** |
| --- | --- | --- |
| **Immune system** | 13 | 212 |
| **Endocrine system** | 9 | 182 |
| **Circulatory system** | 2 | 43 |
| **Digestive system** | 9 | 119 |
| **Excretory system** | 5 | 59 |
| **Nervous system** | 10 | 229 |
| **Sensory system** | 4 | 21 |
| **Development** | 3 | 53 |
| **Environmental adaptation** | 5 | 39 |

***Table S9 Glycoside hydrolases identified in*** A. besseyi

| **CAZy family** | **Pfam domains** | ***A. besseyi* contigs** | **Species** |
| --- | --- | --- | --- |
| **GH 16** | Acyl_transf_3 | 90 | *Streptomyces griseus* |
| **GH 20** | Glyco_hydro_20b|Glyco_hydro_20 | 2 | *Rattus norvegicus* |
| **GH 22** | Destabilase | 2 | *C. briggsae* |
| **GH 30** | Glyco_hydro_30 | 6 | *Phytophthora infestans, C. elegans* |
| **GH 37** | Trehalase | 14 | *C. briggsae* |
| **GH 45** | Glyco_hydro_45 | 4 | *Bursaphelenchus xylophilus* |
| **GH 47** | Glyco_hydro_47 | 10 | *Bos taurus, C. briggsae, Xenopus laevis* |
| **GH56** | Glyco_hydro_56 | 3 | *C. elegans* |
| **GH114** | DUF297 | 4 | *Frankia sp.* |
| **GH117** | Sulfatase | 1 | *Flavobacteriales bacterium* |

***Table S10 Glycosyl transferases identified in*** A. besseyi

| **CAZy family** | **Pfam domains** | 1. ***besseyi***   **contigs** | **Species** |
| --- | --- | --- | --- |
| [**GT1**](http://www.cazy.org/fam/GT1.html) | DAGAT/Glyco_tran_28_C/UDPGT | 105 | *Aspergillus nidulans, Ictalurus furcatus, Papio Anubis, Mus musculus, Aedes aegypti* |
| [**GT2**](http://www.cazy.org/fam/GT2.html) | Glycos_transf_2/Glyco_trans_2_3/ | 6 | *Glossina morsitans morsitans, Glossina morsitans morsitans* |
| [**GT3**](http://www.cazy.org/fam/GT3.html) | Glycogen_syn | 2 | *Steinernema feltiae* |
| [**GT4**](http://www.cazy.org/fam/GT4.html) | Glycos_transf_1 | 4 | *Pongo abelii, Taeniopygia guttata* |
| [**GT7**](http://www.cazy.org/fam/GT7.html) | Glyco_transf_7N|Glyco_transf_7C | 2 | *Caenorhabditis elegans, Trichoplusia ni* |
| [**GT8**](http://www.cazy.org/fam/GT8.html) | Glyco_transf_8 | 12 | *Caenorhabditis elegans* |
| [**GT10**](http://www.cazy.org/fam/GT10.html) | Glyco_transf_10 | 28 | *Apis mellifera carnica/Caenorhabditis elegans/* |
| [**GT13**](http://www.cazy.org/fam/GT13.html) | GNT-I | 5 | *Sus scrofa/Danio rerio* |
| [**GT14**](http://www.cazy.org/fam/GT14.html) | Branch | 7 | *Caenorhabditis elegans* |
| [**GT16**](http://www.cazy.org/fam/GT16.html) | MGAT2 | 3 | *Caenorhabditis elegans* |
| [**GT20**](http://www.cazy.org/fam/GT20.html) | Glyco_transf_20 | 3 | *Aphelenchus avenae* |
| [**GT22**](http://www.cazy.org/fam/GT22.html) | Glyco_transf_22 | 7 | *Xenopus (Silurana) tropicalis, Caenorhabditis brenneri* |
| [**GT24**](http://www.cazy.org/fam/GT24.html) | UDP-g_GGTase | 1 | *Danio rerio* |
| [**GT25**](http://www.cazy.org/fam/GT25.html) | Glyco_transf_25 | 9 | *Caenorhabditis elegans* |
| **GT27** | Glycos_transf_2|Ricin_B_lectin | 8 | *Caenorhabditis elegans, Bos taurus* |
| [**GT31**](http://www.cazy.org/fam/GT31.html) | Fringe/Galactosyl_T | 14 | *Pongo abelii, Plutella xylostella, Lepeophtheirus salmonis* |
| [**GT34**](http://www.cazy.org/fam/GT34.html) | p450/TGT | 28 | *Aspergillus nidulans, Leptosphaeria maculans* |
| [**GT43**](http://www.cazy.org/fam/GT43.html) | Glyco_transf_43 | 6 | *Caenorhabditis briggsae* |
| **GT47/GT64** | Exostosin/Glyco_transf_64 | 9 | *Gallus gallus, Xenopus laevis* |
| [**GT49**](http://www.cazy.org/fam/GT49.html) | Glyco_transf_49 | 5 | *Caenorhabditis elegans* |
| [**GT54**](http://www.cazy.org/fam/GT54.html) | Glyco_transf_54 | 2 | *Rattus norvegicus* |
| [**GT58**](http://www.cazy.org/fam/GT58.html) | ALG3 | 2 | *Caenorhabditis elegans* |
| [**GT65**](http://www.cazy.org/fam/GT65.html) | O-FucT | 6 | *Drosophila pseudoobscura, Drosophila yakuba* |
| [**GT66**](http://www.cazy.org/fam/GT66.html) | STT3 | 29 | *Mus musculus* |
| [**GT68**](http://www.cazy.org/fam/GT68.html) | O-FucT | 2 | *Caenorhabditis elegans* |
| [**GT76**](http://www.cazy.org/fam/GT76.html) | Mannosyl_trans2 | 4 | *Tribolium castaneum* |
| [**GT92**](http://www.cazy.org/fam/GT92.html) | Glyco_transf_92 | 8 | *Nematostella vectensis, Anopheles gambiae, Cryptosporidium parvum* |
| [**GT90**](http://www.cazy.org/fam/GT90.html) | Glyco_transf_90 | 11 | *Brugia malayi, Mus musculus, Xenopus laevis* |

**Table S11 Carbohydrate esterases identified in *A. besseyi***

| **CAZy family** | **Pfam domains** | ***A. besseyi* contigs** | **Species** |
| --- | --- | --- | --- |
| **CE1** | Esterase | 2 | *C. elegans* |
| **CE10** | COesterase/DPPIV_N|Peptidase_S9/Peptidase_S9 | 40 | *Aphis gossypii, C. briggsae, Nilaparvata lugens, Bos taurus, Dictyocaulus viviparus* |

**Table S12 Carbohydrate binding modules identified in *A. besseyi***

| **CAZy family** | **Pfam domains** | ***A. besseyi* contigs** | **Species** |
| --- | --- | --- | --- |
| **CBM5** | Trypsin/Peptidase_S8 | 3 | *Vibrio parahaemolyticus*, *Pseudoalteromonas piscicida* |
| **CBM13** | Astacin | 12 | *Elizabethkingia meningoseptica* |
| **CBM50** | Pkinase | 3 | *Oryza sativa Indica* |

**Table S13 Candidate effectors or potential parasitism genes identified in *A. besseyi***

| **Effector** | **(Speculative) Function** | **Contigs with hit** | **Bit score** | **Best E-value** |
| --- | --- | --- | --- | --- |
| **10A06 = RING-H2** | indirect induction of antioxidant genes in syncytium | 15 | 55 | 1e-07 |
| **14-3-3b** | unknown | 3 | 381 | 1e-106 |
| **2G02** | unknown | 4 | 47 | 2e-5 |
| **4D01** | unknown | 21 | 135 | 2e-32 |
| **4D03** | unknown | 15 | 134 | 6e-32 |
| **5G05** | unknown | 6 | 175 | 5e-44 |
| **6F06** | unknown | 56 | 66 | 5e-11 |
| **acid phosphatase** | digestion | 30 | 324 | 1e-88 |
| **aminopeptidase** | protein degradation | 38 |  | 0 |
| **annexin** | mimicking plant annexin: protection of cells against stress | 4 | 248 | 9e-66 |
| **aspartyl protease** | protein degradation | 6 | 207 | 5e-54 |
| **calcium dependent protein kinase** | involved in giant cell formation | 30 | 567 | 1e-161 |
| **calreticulin** | calcium signalling | 24 | 617 | 1e-177 |
| **cathepsin / cysteine proteinase** | protein degradation | 2 | 449 | 1e-126 |
| **CDC48-like protein** | involved in giant cell formation | 2 | 1357 | 0 |
| **chitinase** | egg hatching | 7 | 125 | 9e-29 |
| **C-type lectin** | unknown | 3 | 48 | 4e-5 |
| **cyclophilins** | chaperone/protein folding to activate effectors | 2 | 479 | 1e-135 |
| **cytokinin** | activation of cell cycle | 30 | 162 | 8e-40 |
| **fatty acid and retinol binding protein (FAR)** | binding of host fatty acids reducing defense response | 12 | 177 | 4e-45 |
| **G8A07** | unknown | 4 | 129 | 5e-29 |
| **galectin** | unknown | 15 | 479 | 1e-135 |
| **glutathione peroxidase** | detoxification of ROS | 10 | 360 | 1e-100 |
| **glutathione-S-transferase** | detoxification of ROS | 57 | 188 | 3e-48 |
| **NAC protein** | involved in cell proliferation | 4 | 239 | 1e-63 |
| **NULG1a** | unknown | 126 | 82 | 8e-16 |
| **peroxiredoxin** | detoxification of ROS | 2 | 50 | 2e-5 |
| **protein disulfide isomerase (PDI)** | protein folding | 4 | 706 | 0 |
| **SKP-1** | involved in signal transduction | 6 | 103 | 3e-22 |
| **SPRYSECs** | plant defense suppression, potential avirulence genes | 2 | 79 | 6e-15 |
| **superoxide dismutase** | detoxification of ROS | 2 | 131 | 2e-31 |
| **SXP-RAL2** | unknown | 2 | 105 | 1e-23 |
| **thioredoxin** | detoxification of ROS | 11 | 522 | 1e-148 |
| **translationally controlled tumor protein (TCTP)** | involved in giant cell formation | 4 | 281 | 3e-76 |
| **transthyretin-like proteins** | growth regulation | 65 | 179 | 1e-45 |
| **triosephosphate isomerase (TPI)** | function in metabolism | 4 | 390 | 1e-109 |
| **ubiquitin carboxyl-terminal hydrolase** | selective protein degradation | 2 | 232 | 1e-60 |
| **ubiquitin extension protein** | selective protein degradation | 54 | 136 | 4e-33 |
| **ubiquitin-activating enzyme** | selective protein degradation | 14 | 1216 | 0 |
| **ubiquitin-like protein** | selective protein degradation | 6 | 246 | 3e-66 |
| **venom allergen proteins** | recognition between plant and nematode | 34 | 100 | 1e-21 |

**Table S14** Small RNA biosynthesis proteins

| ***C. elegans* orthologs** | ***A. besseyi* contigs *** | **Highest Score (bits)** | **E-value** |
| --- | --- | --- | --- |
| **drh-3** | 23 | 303 | 6e-082 |
| **drsh-1a** | 7 | 534 | 1e-151 |
| **drsh-1b** | 7 | 533 | 1e-151 |
| **xpo-1a** | 4 | 1108 | 0.0 |
| **xpo-1b** | 4 | 95 | 2e-020 |
| **xpo-2** | 4 | 499 | 1e-141 |
| **dcr-1** | 35 | 686 | 0.0 |
| **drh-1** | 26 | 249 | 1e-065 |
| **pash-1** | 2 | 162 | 1e-039 |

*****(bitscore>40 and E-value<0.0001)

**Table S15** dsRNA uptake and spreading effectors

|  | ***C. elegans* orthologs** | ***A. besseyi* contigs *** | **Highest Score (bits)** | **E-value** |
| --- | --- | --- | --- | --- |
| **Amplification Proteins** | |  |  |  |
|  | smg-2 | 30 | 932 | 0.0 |
|  | smg-6a | 3 | 67 | 1e-010 |
|  | ego-1 | 10 | 746 | 0.0 |
|  | rrf-3 | 12 | 481 | 1e-135 |
|  | rrf-1a | 10 | 724 | 0.0 |
|  | rrf-1b | 10 | 724 | 0.0 |
| **Spreading Proteins** |  |  |  |  |
|  | rsd-3 | 9 | 267 | 2e-071 |

*****(bitscore>40 and E-value<0.0001)

**Table S16** Argonautes and RNA-induced silencing complex components

|  | ***C. elegans* orthologs** | ***A. besseyi* contigs *** | **Highest Score (bits)** | **E-value** |
| --- | --- | --- | --- | --- |
| **Argonautes** |  |  |  |  |
|  | alg-1a | 59 | 1471 | 0.0 |
|  | alg-1b | 57 | 1471 | 0.0 |
|  | R06C7.1 | 95 | 332 | 2e-090 |
|  | C04F12.1 | 60 | 171 | 4e-042 |
|  | F58G1.1 | 120 | 304 | 3e-082 |
|  | alg-4 | 115 | 979 | 0.0 |
|  | rde-1 | 64 | 189 | 1e-047 |
|  | C16C10.3 | 108 | 188 | 3e-047 |
|  | ppw-1a | 41 | 207 | 3e-053 |
|  | ppw-1c | 37 | 207 | 3e-053 |
|  | ppw-1d | 36 | 193 | 6e-049 |
|  | csr-1a | 57 | 201 | 3e-051 |
|  | csr-1b | 49 | 201 | 2e-051 |
|  | ppw-2 | 119 | 299 | 9e-081 |
|  | T22B3.2a | 103 | 968 | 0.0 |
|  | T22B3.2b | 103 | 968 | 0.0 |
|  | T22H9.3 | 103 | 205 | 2e-052 |
|  | alg-2a | 57 | 1387 | 0.0 |
|  | alg-2b | 57 | 1387 | 0.0 |
|  | ergo-1 | 47 | 191 | 4e-048 |
|  | prg-1 | 37 | 131 | 4e-030 |
|  | T23D8.7 | 57 | 613 | e-175 |
|  | nrde-3 | 77 | 172 | 2e-042 |
|  | sago-2a | 36 | 211 | 3e-054 |
|  | sago-2b | 36 | 208 | 2e-053 |
|  | T23B3.2 | 22 | 70 | 4e-013 |
|  | Y49F6A.1 | 113 | 199 | 2e-050 |
|  | ZK1248.7 | 131 | 329 | 8e-090 |
|  | prg-2 | 34 | 124 | 4e-028 |
|  | C14B1.7 | 113 | 177 | 5e-044 |
| **RISC Proteins** | |  |  |  |
|  | tsn-1 | 2 | 936 | 0.0 |
|  | ain-1 | 10 | 79 | 2e-014 |
|  | vig-1a | 12 | 164 | 1e-040 |
|  | ain-2a | 14 | 65 | 2e-010 |
|  | ain-2b | 9 | 67 | 8e-011 |
|  | ain-2c | 14 | 66 | 2e-010 |
|  | ain-2d | 8 | 47 | 2e-007 |

*****(bitscore>40 and E-value<0.0001)

**Table S17** RNAi inhibitors

| ***C.elegans* orthologs** | ***A. besseyi* contigs *** | **Highest Score (bits)** | **E-value** |
| --- | --- | --- | --- |
| **xrn-2** | 37 | 1077 | 0.0 |
| **adr-2** | 2 | 100 | 6e-021 |
| **xrn-1** | 8 | 435 | 1e-121 |
| **adr-1** | 2 | 44 | 5e-005 |
| **eri-7** | 24 | 191 | 3e-048 |

*****(bitscore>40 and E-value<0.0001)

**Table S18** Nuclear effectors

| ***C.elegans*orthologs** | ***A. besseyi* contigs *** | **Highest Score (bits)** | **E-value** |
| --- | --- | --- | --- |
| **mut-7** | 10 | 84 | 1e-015 |
| **cid-1** | 14 | 193 | 1e-048 |
| **ekl-1** | 2 | 91 | 5e-018 |
| **gfl-1** | 6 | 183 | 1e-046 |
| **mes-2** | 54 | 272 | 9e-073 |
| **ekl-4a** | 17 | 278 | 7e-075 |
| **ekl-4b** | 17 | 278 | 8e-075 |
| **mes-6** | 85 | 160 | 4e-039 |
| **rha-1** | 81 | 1056 | 0.0 |
| **ekl-6** | 3 | 107 | 8e-023 |
| **zfp-1a** | 9 | 129 | 1e-029 |
| **zfp-1c** | 2 | 47 | 5e-005 |
| **mut-2a** | 14 | 103 | 4e-022 |
| **mut-2b** | 16 | 91 | 1e-018 |
| **mut-16a** | 44 | 64 | 9e-010 |
| **mut-16b** | 44 | 64 | 9e-010 |

**Table S19** Neuropeptide genes encoding insulin-like peptides

|  | **Cotings** | **Score** | **Brest E value** | **signal peptide** |
| --- | --- | --- | --- | --- |
| **daf-28** | 2 | 39 | 0.001 | Yes |
| **ins-1** | 5 | 55 | 2e-008 | No |
| **ins-6** | 2 | 39 | 0.002 | Yes |
| **ins-7** | 2 | 39 | 9e-004 | Yes |
| **ins-8** | 2 | 43 | 8e-005 | No |
| **ins-17** | 5 | 76 | 8e-015 | No |
| **ins-18** | 5 | 67 | 4e-012 | Yes |
| **ins-20** | 2 | 25 | 0.003 | No |

**Table S20** Neuropeptide genes encoding FMRFamide-related peptides

|  | **Coting** | **Score** | **Brest E value** | **Signal peptide** | |
| --- | --- | --- | --- | --- | --- |
| **flp-1** | 4 | 82 | 3e-016 | Yes |  |
| **flp-2** | 2 | 44 | 5e-005 | No |  |
| **flp-3** | 6 | 82 | 4e-016 | No |  |
| **flp-4** | 2 | 49 | 2e-006 | Yes |  |
| **flp-5** | 2 | 45 | 2e-005 | No |  |
| **flp-6** | 2 | 104 | 5e-023 | Yes |  |
| **flp-7** | 4 | 109 | 1e-024 | Yes |  |
| **flp-8** | 2 | 48 | 3e-006 | Yes |  |
| **flp-11** | 4 | 91 | 2e-019 | Yes |  |
| **flp-12** | 2 | 65 | 1e-011 | Yes |  |
| **flp-13** | 4 | 67 | 6e-012 | No |  |
| **flp-14** | 6 | 51 | 5e-007 | No |  |
| **flp-16** | 2 | 64 | 4e-011 | Yes |  |
| **flp-17** | 4 | 61 | 3e-010 | Yes |  |
| **flp-18** | 4 | 121 | 6e-028 | Yes |  |
| **flp-21** | 2 | 46 | 7e-006 | Yes |  |
| **flp-22** | 2 | 56 | 7e-009 | Yes |  |

**Table S21** Neuropeptide genes encoding non-Insulin, non-FLP Peptides

|  | **Coting** | **Score** | **BrestEvalue** |
| --- | --- | --- | --- |
| **nlp-2** | 4 | 64 | 1e-010 |
| **nlp-4** | 2 | 49 | 2e-006 |
| **nlp-9** | 4 | 51 | 3e-007 |
| **nlp-10** | 2 | 56 | 9e-009 |
| **nlp-12** | 3 | 75 | 2e-014 |
| **nlp-13** | 4 | 57 | 4e-009 |
| **nlp-14** | 4 | 136 | 2e-032 |
| **nlp-15** | 5 | 90 | 7e-019 |
| **nlp-16** | 12 | 52 | 7e-007 |
| **nlp-21** | 4 | 45 | 3e-005 |
| **nlp-24** | 28 | 55 | 2e-008 |
| **nlp-25** | 10 | 48 | 2e-006 |
| **nlp-26** | 23 | 52 | 1e-007 |
| **nlp-27** | 22 | 60 | 6e-010 |
| **nlp-28** | 15 | 52 | 1e-007 |
| **nlp-29** | 16 | 55 | 2e-008 |
| **nlp-30** | 23 | 56 | 9e-009 |
| **nlp-31** | 46 | 57 | 5e-009 |
| **nlp-32** | 21 | 54 | 4e-008 |
| **nlp-33** | 22 | 64 | 4e-011 |
| **nlp-34** | 12 | 52 | 2e-007 |
| **nlp-36** | 2 | 51 | 2e-007 |
| **nlp-37** | 2 | 55 | 2e-008 |
| **nlp-40** | 2 | 73 | 6e-014 |

**Table S22 Peptidases identified in *A. besseyi***

| **Family** | **E value** | **Peptidase** | **Species** |
| --- | --- | --- | --- |
| **A22A** | 1.50e-106 | SEL-12 peptidase | *Caenorhabditis elegans* |
| **A22B** | 1.10e-60 | impas 1 peptidase | *Homo sapiens* |
| **C02A** | 1.60e-108 | calpain tra-3 | *Caenorhabditis elegans* |
| **C12** | 8.10e-80 | ubiquitinyl hydrolase-L5 | *Brugia malayi* |
| **C13** | 2.40e-96 | glycosylphosphatidylinositol: protein transamidase | *Bos taurus* |
| **C14B** | 2.00e-30 | paracaspase | *Homo sapiens* |
| **C15** | 3.40e-21 | pyroglutamyl-peptidase I (chordate) | *Homo sapiens* |
| **C19** | 1.10e-14 | family C19 unassigned peptidases | *Xenopus laevis* |
| **C26** | 7.80e-70 |  |  |
| **C44** | 5.60e-15 | asparagine synthetase | *Homo sapiens* |
| **C46** | 4.20e-18 | Tiggy-winkle protein | *Brachydanio rerio* |
| **C48** | 3.50e-16 | Ulp2 peptidase | *Saccharomyces cerevisiae* |
| **C50** | 2.10e-47 | SEP-1 peptidase | *Caenorhabditis elegans* |
| **C54** | 1.80e-73 | autophagin-1 | *Homo sapiens* |
| **C56** | 2.10e-26 | KIAA0361 protein | *Homo sapiens* |
| **C64** | 2.80e-71 | Cezanne deubiquitinylating peptidase | *Homo sapiens* |
| **C65** | 1.60e-46 | otubain-1 | *Homo sapiens* |
| **C78** | 1.30e-69 | UfSP2 peptidase | *Mus musculus* |
| **C85** | 5.30e-39 | OTLD1 deubiquitinylating enzyme | *Homo sapiens* |
| **C86** | 2.50e-24 | Josephin domain containing 2 | *Homo sapiens* |
| **C89** | 9.60e-64 | acid ceramidase precursor | *Homo sapiens* |
| **I02** | 1.40e-13 | tissue factor pathway inhibitor-2 unit 3 | *Mus musculus* |
| **I04** | 1.10e-55 | serpin srp-2 | *Caenorhabditis elegans* |
| **I21** | 8.00e-15 | secretogranin V | *Homo sapiens* |
| **I25B** | 1.50e-15 | cystatin Bm-CPI-2 | *Brugia malayi* |
| **I29** | 2.70e-44 | cathepsin L propeptide | *Homo sapiens* |
| **I51** | 1.20e-27 | phosphatidylethanolamine-binding protein | *Homo sapiens* |
| **M01** | 8.50e-28 | ERAP2 aminopeptidase | *Homo sapiens* |
| **M02** | 2.00e-87 | angiotensin-converting enzyme peptidase unit 2 | *Homo sapiens* |
| **M03A** | 4.60e-17 | mitochondrial intermediate peptidase | *Oryzias latipes* |
| **M08** | 1.20e-08 | family M8 non-peptidase homologues | *Trypanosoma cruzi* |
| **M10A** | 2.80e-20 | karilysin | *Tannerella forsythia* |
| **M12A** | 1.90e-18 | myosinase | *Todarodes pacificus* |
| **M12B** | 1.50e-36 | ADAM19 peptidase | *Mus musculus* |
| **M13** | 5.40e-11 | Nep2 peptidase (insect) | *Tribolium castaneum* |
| **M14A** | 3.20e-13 | suro-1 g.p. | *Caenorhabditis briggsae* |
| **M14B** | 7.90e-91 | metallocarboxypeptidase D peptidase unit 2 | *Anas platyrhynchos* |
| **M16B** | 1.40e-54 | subfamily M16B non-peptidase homologues | *Ixodes scapularis* |
| **M16C** | 2.50e-59 | insulysin homologue | *Cryptococcus neoformans* |
| **M17** | 2.50e-98 | leucyl aminopeptidase-1 ({Caenorhabditis}-type) | *Homo sapiens* |
| **M18** | 2.20e-99 | At5g60160 -like peptidase | *Arabidopsis thaliana* |
| **M20A** | 8.80e-52 | subfamily M20A unassigned peptidases | *Gibberella zeae* |
| **M22** | 2.40e-15 | family M22 non-peptidase homologues | *Thermotoga lettingae* |
| **M24A** | 1.50e-148 | methionyl aminopeptidase 2 | *Homo sapiens* |
| **M24B** | 2.20e-65 | app-1 g.p. | *Caenorhabditis elegans* |
| **M24X** | 8.90e-56 | chromatin-specific transcription elongation factor 140 kDa subunit | *Homo sapiens* |
| **M28B** | 6.20e-11 | glutamate carboxypeptidase II | *Homo sapiens* |
| **M28X** | 7.30e-43 | Mername-AA103 peptidase | *Homo sapiens* |
| **M38** | 1.30e-97 | 1300019j08rik protein | *Homo sapiens* |
| **M41** | 6.20e-47 | AtFtsH11 peptidase | *Arabidopsis thaliana* |
| **M48A** | 7.00e-81 | farnesylated-protein converting enzyme 1 | *Homo sapiens* |
| **M50A** | 2.00e-39 | S2P peptidase | *Homo sapiens* |
| **M67A** | 1.20e-14 | COP9 signalosome subunit 6 | *Homo sapiens* |
| **M67X** | 9.20e-43 | eukaryotic translation initiation factor 3 subunit 5 | *Homo sapiens* |
| **S01A** | 2.60e-12 | mannan-binding lectin-associated serine peptidase 1 | *Rattus norvegicus* |
| **S01X** | 3.40e-32 | family S1 unassigned peptidases | *Caenorhabditis briggsae* |
| **S08B** | 7.30e-79 | PCSK1 peptidase | *Homo sapiens* |
| **S09A** | 9.60e-100 | prolyl oligopeptidase | *Sus scrofa* |
| **S09B** | 1.10e-57 | DPF-3 peptidase | *Drosophila melanogaster* |
| **S09C** | 4.00e-58 | acylaminoacyl-peptidase | *Homo sapiens* |
| **S09X** | 9.50e-46 | BC026374 protein | *Mus musculus* |
| **S10** | 1.10e-10 | BRS1 serine carboxypeptidase | *Arabidopsis thaliana* |
| **S12** | 9.90e-29 | LACT-1 peptidase | *Mus musculus* |
| **S14** | 1.80e-73 | peptidase Clp (type 3) | *Homo sapiens* |
| **S16** | 4.10e-49 | psLon peptidase | *Homo sapiens* |
| **S26A** | 1.50e-19 | mitochondrial inner membrane peptidase 1 | *Schizosaccharomyces pombe* |
| **S26B** | 2.00e-42 | signalase (animal) 18 kDa component | *Canis familiaris* |
| **S28** | 8.80e-45 | CG9953 protein ({Drosophila melanogaster}) | *Tribolium castaneum* |
| **S33** | 1.20e-68 | At4g10050 | *Arabidopsis thaliana* |
| **S54** | 2.60e-35 | PARL peptidase | *Homo sapiens* |
| **S59** | 9.30e-38 | nucleoporin 145 | *Homo sapiens* |
| **T01A** | 4.90e-46 | proteasome catalytic subunit 2i | *Homo sapiens* |
| **T02** | 1.90e-36 | glycosylasparaginase precursor | *Homo sapiens* |
| **T03** | 5.50e-202 | gamma-glutamyltransferase | *Brugia malayi* |
| **U48** | 2.10e-46 | fce-2 g.p. | *Caenorhabditis elegans* |

**Text S1 Detailed methods of RNAseq libraries construction**

Polyadenylated RNA was enriched by two rounds of poly-A selection with poly-T oligo-attached magnetic beads. The RNA was then chemically fragmented, treated with Antarctic phosphatase (NEB, Herts, UK) and subsequently with polynucleotide kinase (NEB, Herts, UK). V1.5 sRNA 3`Adapter was ligated to the RNA with T4 RNA ligase 2 truncated (NEB, Herts, UK), and SRA 5` Adapter was ligated to the RNA with T4 RNA Ligase. After ligation of the SRA RT primer, the RNA was reverse transcribed with SuperScript II Reverse Transcriptase (Invitrogen, Carlsbad, CA, USA). Double stranded sequencing library DNA was then produced by 15 cycles PCR with primers GX1 and GX2. The PCR products from 200bp to 300bp were recycled by 6% polyacrylamide Tris-borate-EDTA gel.

**Text S2** **MIRA assembly yielded sequences were not found in our dataset**

Ab_MIRA_c2366, Ab_MIRA_c2650, Ab_MIRA_c3590, Ab_MIRA_c3615, Ab_MIRA_c4689, Ab_MIRA_c4914, Ab_MIRA_c5094, Ab_MIRA_c5158, Ab_MIRA_rep_c1010, Ab_MIRA_rep_c1222, Ab_MIRA_rep_c1394, Ab_MIRA_rep_c1491, Ab_MIRA_rep_c1816, Ab_MIRA_rep_c1839, Ab_MIRA_rep_c1908, Ab_MIRA_rep_c2032, Ab_MIRA_rep_c2154, Ab_MIRA_rep_c2674, Ab_MIRA_rep_c2684, Ab_MIRA_rep_c2866, Ab_MIRA_rep_c2910, Ab_MIRA_rep_c2991, Ab_MIRA_rep_c3019, Ab_MIRA_rep_c3121, Ab_MIRA_rep_c3238, Ab_MIRA_rep_c3311, Ab_MIRA_rep_c3664, Ab_MIRA_rep_c3760, Ab_MIRA_rep_c3770, Ab_MIRA_rep_c3802, Ab_MIRA_rep_c3824, Ab_MIRA_rep_c3879, Ab_MIRA_rep_c4114, Ab_MIRA_rep_c4231, Ab_MIRA_rep_c4270, Ab_MIRA_rep_c4370, Ab_MIRA_rep_c4386, Ab_MIRA_rep_c4528, Ab_MIRA_rep_c4561, Ab_MIRA_rep_c4608, Ab_MIRA_rep_c4611, Ab_MIRA_rep_c4631, Ab_MIRA_rep_c4732, Ab_MIRA_rep_c4810, Ab_MIRA_rep_c4821, Ab_MIRA_rep_c4843, Ab_MIRA_rep_c4917, Ab_MIRA_rep_c4984, Ab_MIRA_rep_c4997, Ab_MIRA_rep_c5017, Ab_MIRA_rep_c5020, Ab_MIRA_rep_c5064, Ab_MIRA_rep_c5069, Ab_MIRA_rep_c5075, Ab_MIRA_rep_c5104, Ab_MIRA_rep_c5127, Ab_MIRA_rep_c5130, Ab_MIRA_rep_c5204, Ab_MIRA_rep_c5266, Ab_MIRA_rep_c5283, Ab_MIRA_rep_c5292, Ab_MIRA_rep_c5324, Ab_MIRA_rep_c5328, Ab_MIRA_rep_c5335, Ab_MIRA_rep_c5338, Ab_MIRA_rep_c5345, Ab_MIRA_rep_c5374, Ab_MIRA_rep_c5395, Ab_MIRA_rep_c5403, Ab_MIRA_rep_c5412, Ab_MIRA_rep_c5416, Ab_MIRA_rep_c5417, Ab_MIRA_rep_c5424, Ab_MIRA_rep_c5428, Ab_MIRA_rep_c5433, Ab_MIRA_rep_c5435, Ab_MIRA_rep_c5439, Ab_MIRA_rep_c5447, Ab_MIRA_rep_c5459, Ab_MIRA_rep_c5464, Ab_MIRA_rep_c5472, Ab_MIRA_rep_c5484, Ab_MIRA_rep_c5492, Ab_MIRA_rep_c5517, Ab_MIRA_rep_c5533, Ab_MIRA_rep_c5538, Ab_MIRA_rep_c5540, Ab_MIRA_rep_c5553, Ab_MIRA_rep_c5564, Ab_MIRA_rep_c5569, Ab_MIRA_rep_c5574, Ab_MIRA_rep_c5578, Ab_MIRA_rep_c5581, Ab_MIRA_rep_c5583, Ab_MIRA_rep_c5585, Ab_MIRA_rep_c5588, Ab_MIRA_rep_c5590, Ab_MIRA_rep_c5591, Ab_MIRA_rep_c5592, Ab_MIRA_rep_c5596, Ab_MIRA_rep_c5598, Ab_MIRA_rep_c5601, Ab_MIRA_rep_c5604, Ab_MIRA_rep_c5606, Ab_MIRA_rep_c5610, Ab_MIRA_rep_c5612, Ab_MIRA_rep_c5616, Ab_MIRA_rep_c5617, Ab_MIRA_rep_c5619, Ab_MIRA_rep_c5622, Ab_MIRA_rep_c5623, Ab_MIRA_rep_c5637, Ab_MIRA_rep_c5638, Ab_MIRA_rep_c5644, Ab_MIRA_rep_c5646, Ab_MIRA_rep_c5649, Ab_MIRA_rep_c5658, Ab_MIRA_rep_c5659, Ab_MIRA_rep_c5660, Ab_MIRA_rep_c5666, Ab_MIRA_rep_c5668, Ab_MIRA_rep_c5686, Ab_MIRA_rep_c5696, Ab_MIRA_rep_c5799, Ab_MIRA_rep_c980
